# The gut microbiota as an early predictor of COVID-19 severity

**DOI:** 10.1128/msphere.00181-24

**Published:** 2024-09-19

**Authors:** Marco Fabbrini, Federica D’Amico, Bernardina T. F. van der Gun, Monica Barone, Gabriele Conti, Sara Roggiani, Karin I. Wold, María F. Vincenti-Gonzalez, Gerolf C. de Boer, Alida C. M. Veloo, Margriet van der Meer, Elda Righi, Elisa Gentilotti, Anna Górska, Fulvia Mazzaferri, Lorenza Lambertenghi, Massimo Mirandola, Maria Mongardi, Evelina Tacconelli, Silvia Turroni, Patrizia Brigidi, Adriana Tami

**Affiliations:** 1 Unit of Microbiome Science and Biotechnology, Department of Pharmacy and Biotechnology, University of Bologna, Bologna, Italy; 2 Human Microbiomics Unit, Department of Medical and Surgical Sciences, University of Bologna, Bologna, Italy; 3 Department of Medical Microbiology and Infection Prevention, University of Groningen, University Medical Center Groningen, Groningen, the Netherlands; 4 Spatial Epidemiology Lab (SpELL), Université Libre de Bruxelles (ULB), Brussels, Belgium; 5 Department of Diagnostics and Public Health, Infectious Diseases Department, University of Verona, Verona, Italy; University of Michigan, Ann Arbor, Michigan, USA

**Keywords:** gut microbiota, COVID-19 severity, machine learning

## Abstract

Several studies reported alterations of the human gut microbiota (GM) during COVID-19. To evaluate the potential role of the GM as an early predictor of COVID-19 at disease onset, we analyzed gut microbial samples of 315 COVID-19 patients that differed in disease severity. We observed significant variations in microbial diversity and composition associated with increasing disease severity, as the reduction of short-chain fatty acid producers such as *Faecalibacterium* and *Ruminococcus*, and the growth of pathobionts as *Anaerococcus* and *Campylobacter*. Notably, we developed a multi-class machine-learning classifier, specifically a convolutional neural network, which achieved an 81.5% accuracy rate in predicting COVID-19 severity based on GM composition at disease onset. This achievement highlights its potential as a valuable early biomarker during the first week of infection. These findings offer promising insights into the intricate relationship between GM and COVID-19, providing a potential tool for optimizing patient triage and streamlining healthcare during the pandemic.

**IMPORTANCE:**

Efficient patient triage for COVID-19 is vital to manage healthcare resources effectively. This study underscores the potential of gut microbiota (GM) composition as an early biomarker for COVID-19 severity. By analyzing GM samples from 315 patients, significant correlations between microbial diversity and disease severity were observed. Notably, a convolutional neural network classifier was developed, achieving an 81.5% accuracy in predicting disease severity based on GM composition at disease onset. These findings suggest that GM profiling could enhance early triage processes, offering a novel approach to optimizing patient management during the pandemic.

## INTRODUCTION

Coronavirus disease 2019 (COVID-19), a respiratory illness caused by severe acute respiratory syndrome coronavirus 2 (SARS-CoV-2), rapidly became a major public health challenge with global implications. The COVID-19 pandemic has been compared to other major historical outbreaks, such as the Spanish flu of 1918 and the Black Death of the 14th century (1). While modern medicine and vaccines helped slow the spread of the virus, the pandemic had nevertheless a significant impact on human health and well-being (2, 3). Early studies suggested a relatively high mortality rate, but this rate has since been revised downward as more information has become available and different variants have emerged. While the lethality rate varies by region and country, it is estimated to be around 0.5%–3% globally, depending on a variety of factors (4). As previously reported (5), after initial exposure to SARS-CoV-2, patients typically develop a diverse range of mild to moderate symptoms within 5–6 days, although it’s worth noting that up to 19.9% of cases may remain asymptomatic (6). Then, if the initial host immune response is unable to control the infection, symptoms generally worsen and may require hospitalization, which usually occurs within the first week after the onset of mild to moderate symptoms (7). Treatment of COVID-19 primarily involves symptomatic relief (e.g., antipyretics, anti-inflammatory drugs, cough suppressants, etc.), oxygen therapy, antiviral medications, or monoclonal antibodies. This underscores the importance of efficiently triaging patients. Several tools have been developed to effectively aid in the COVID-19 triage (8–11), most of which weigh patient symptoms, blood parameters, and imaging. Additionally, in current clinical practice, decisions on therapeutic approaches, such as the administration of antiviral medication, also take into consideration crucial factors including age, patient weight, and comorbidities.

The gut microbiota (GM) has been recognized as a critical factor in immune function and overall health, and several studies have suggested that COVID-19 may cause dysbiotic changes in the gut microbiota (12–14). Such perturbations in the natural gut ecosystem may contribute to the inflammatory response seen in severe cases of the disease, which can lead to complications such as acute respiratory distress syndrome (ARDS) and death. However, most studies have primarily focused on hospitalized severe COVID-19 patients, comparing their gut microbiota composition with that of healthy subjects enrolled before the COVID-19 pandemic or other hospitalized patients who tested negative (12, 14, 15). Oftentimes, severity classes were defined based on center-specific clinical characteristics, especially in earlier works, lacking a common parameter for severity stratification. In an effort to address these knowledge gaps and gain a more comprehensive understanding of COVID-19’s impact on the gut, we conducted a study in which we profiled the gut microbiota of 315 subjects with varying degrees of COVID-19 severity. Additionally, we included a group of healthy, uninfected subjects for comparison, all of whom were enrolled concurrently with the COVID-19 patients (matching the pandemics period). We employed 16S rRNA amplicon sequencing for this analysis. To ensure robust results, we categorized COVID-19 severity based on World Health Organization (WHO) guidelines (16). Our data revealed significant differences in microbiota composition and network structure according to COVID-19 severity. In addition, we tested several machine-learning classifier architectures to prove that microbiota compositional data can indeed be used for preemptive triage of patient severity starting from about 1 week after symptom onset. Our results, while far from being exhaustive, encourage the use of gut microbiota as a potential biomarker to be considered to support clinical decision-making for COVID-19.

## RESULTS

### Study cohort description

**Fig 1:**
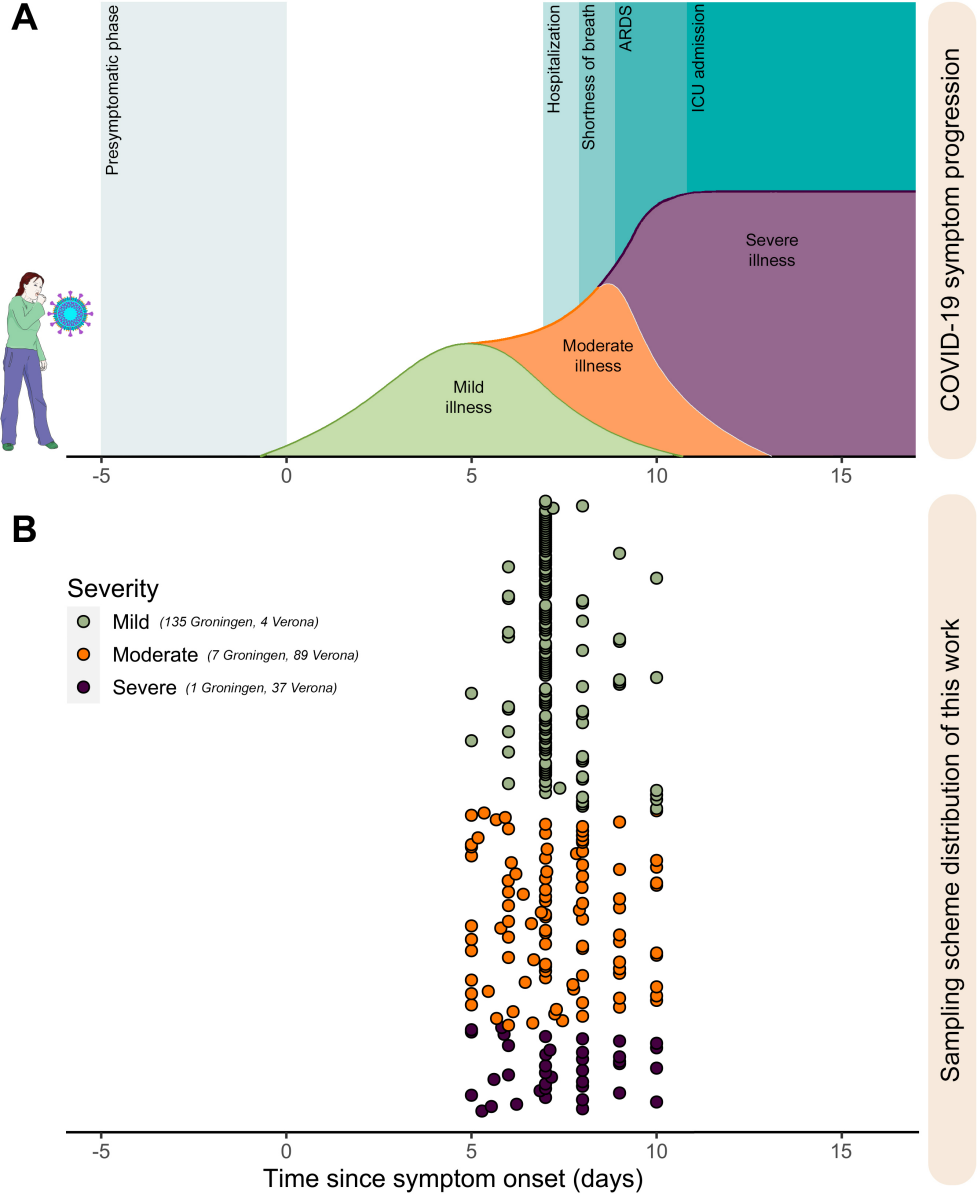
Sampling distribution of recruited COVID-19 patients with different symptom severity and healthy uninfected subjects. In the context of the general symptom onset of COVID-19 (A), fecal samples were collected between the 5th and 10th day after symptom onset. Healthy uninfected controls were household members of individuals who had tested negative for SARS-CoV-2 and showed no symptoms. To maintain correspondence between the positive individuals and controls, uninfected subjects were sampled concurrently with the positive cases. (B) Samples from moderate and severe patients were collected at hospital admission, and the reported time since symptom onset was obtained from medical history. Samples from mild subjects were collected at a similar time (~7 days) from symptom onset to approximate a similar disease progression in inpatients. In addition to the samples presented in the plot, 42 healthy uninfected subjects were considered as well to complete the coverage of the severity classes. Such healthy uninfected subjects were household members of the mild ones and were sampled concomitantly. Therefore, the healthy uninfected subjects were tested SARS-CoV-2 negative via nose swab PCR in the day of sample collection and did not report COVID-19 symptoms. The numerosity of samples from the two recruiting centers (Groningen—the COVID-HOME cohort [17] of the Department of Medical Microbiology and Infection Prevention, University Medical Center Groningen, Groningen, The Netherlands, and Verona—the Infectious Diseases Unit, Department of Diagnostics and Public Health, University of Verona, Verona, Italy) included in each severity group is shown. ICU, intensive care unit. The figure was partly generated using Servier Medical Art, provided by Servier, licensed under a Creative Commons Attribution 3.0 unported license.

This is a multi-center observational cohort study conducted as part of the EU project ORCHESTRA (Connecting European Cohorts to Increase Common and Effective Response to SARS-CoV-2 Pandemic), grant agreement no. 101016167. The recruiting centers involved in this study were (i) the COVID-HOME cohort (17) of the Department of Medical Microbiology and Infection Prevention, University Medical Center Groningen, Groningen, The Netherlands, and (ii) the Infectious Diseases Unit, Department of Diagnostics and Public Health, University of Verona, Verona, Italy. This work included a total of 315 participants, recruited during the first, second, and third waves of the SARS-CoV-2 pandemic. Samples from the COVID-HOME cohort were collected between August 2020 and October 2021, covering an extensive period of the pandemic. Additionally, samples from Verona were collected from April to May 2021. Participants were representative of the full range of COVID-19 severity defined according to the WHO clinical progression scale (16), encompassing healthy uninfected subjects, COVID-19 patients exhibiting signs of mild illness who were at home, and hospitalized moderate, severe, and fatal cases. To balance the numerosity of patients stratified into severity classes, we combined severe and fatal cases into a single “severe” class. The uninfected subjects were healthy and symptom-free individuals who tested negative for SARS-CoV-2 by nose swab quantitative PCR and had not been positive previously and thus were used here as a control group. All subjects were sampled between days 5 and 10 after symptom onset, when the majority of COVID-19 patients had mild to moderate symptoms. The controls were healthy uninfected household members of positive enrolled subjects, sampled at the same time. This allowed us to harmonize sampling at the same stage of disease progression, finally considering patient severity outcome at the end of the infection. In total, the cohort included 42 healthy uninfected subjects, 139 patients who maintained mild COVID-19, 96 with moderate symptoms, and 38 who progressed to severe clinical manifestations, of whom 5 died. The sample distribution across recruiting centers, severity classes (i.e., uninfected, mild, moderate and severe), and sampling timing is detailed in Fig. 1. Further details on the cohort can be found in Table 1.

**TABLE 1:** Study population characteristics at the first week of COVID-19 post-symptom onset^*a*^

Data	Total	Groningen	Verona
General data			
*N*	315	185	130
Age: mean ± SEM (min–max)	46.6 ± 1.2 (0–94)	36.7 ± 1.5 (0–77)	60.6 ± 1.4 (21–94)
Sex: F, M (% F, % M)	148, 167 (47.0%, 53.0%)	103, 82 (55.7%, 44.3%)	45, 85 (34.6%, 65.4%)
BMI	25.7 ± 0.4 (12.7–47.4)	24.4 ± 0.4 (12.7–36.5)	28.3 ± 0.7 (19.5–47.4)
SARS-CoV-2 positivity: +, – (% +, % –)	273, 42 (86.7%, 13.3%)	143, 42 (77.3%, 22.7%)	130, 0 (100%, 0%)
Severity classes (WHO)			
Uninfected	42	42	0
Mild	139	135	4
Moderate	96	7	89
Severe/dead	38	1	37
Comorbidities (positive participants, *N* = 273)			
Cardiovascular: Y (%)	66 (24.4%)	2 (1.4%)	64 (49.2%)
Diabetes: Y (%)	27 (10%)	5 (3.5%)	22 (16.9%)
Chronic respiratory diseases: Y (%)	18 (6.6%)	0 (0%)	18 (13.8%)
Immunosuppressed: Y (%)	11 (4.1%)	0 (0%)	11 (8.5%)
Cancer: Y (%)	10 (3.7%)	6 (4.2%)	4 (3.1%)
Symptoms (positive participants, *N* = 273)			
Abdominal pain: Y (%)	52 (19.2%)	39 (27.3%)	13 (10%)
Vomiting/nausea: Y (%)	50 (18.5%)	31 (21.7%)	19 (14.6%)
Diarrhea: Y (%)	73 (26.9%)	41 (28.7%)	32 (24.6%)
Fever: Y (%)	170 (62.7%)	56 (39.2%)	114 (87.7%)
Cough: Y (%)	175 (64.6%)	94 (65.7%)	81 (62.3%)
Short breath: Y (%)	141 (52%)	54 (37.8%)	87 (66.9%)
Sore throat: Y (%)	89 (32.8%)	79 (55.2%)	10 (7.7%)
Nasal congestion: Y (%)	91 (33.6%)	82 (57.3%)	9 (6.9%)
Fatigue: Y (%)	184 (67.9%)	110 (76.9%)	74 (56.9%)
Ageusia: Y (%)	116 (42.8%)	84 (58.7%)	32 (24.6%)
Anosmia: Y (%)	109 (40.2%)	84 (58.7%)	25 (19.2%)
Skin rash: Y (%)	18 (6.6%)	15 (10.5%)	3 (2.3%)
Viral variants (positive participants, *N* = 174)			
20A: N (%)	22 (8.1%)	22 (15.4%)	0 (0%)
20B: N (%)	1 (0.4%)	1 (0.7%)	0 (0%)
20E (EU1): N (%)	26 (9.5%)	26 (18.2%)	0 (0%)
Alpha-20I: N (%)	83 (30.4%)	65 (45.5%)	18 (13.8%)
Delta-21I: N (%)	2 (0.7%)	0 (0%)	2 (1.5%)
Delta-21J: N (%)	40 (14.7%)	0 (0%)	40 (30.8%)

^*a*^ F, female; M, male; BMI, body mass index; SEM, standard error of the mean; WHO, World Health Organization. The viral variants lister are indeed viral clade assigned through Nextclade.

### COVID-19 severity-related changes in gut microbiota composition

**Fig 2:**
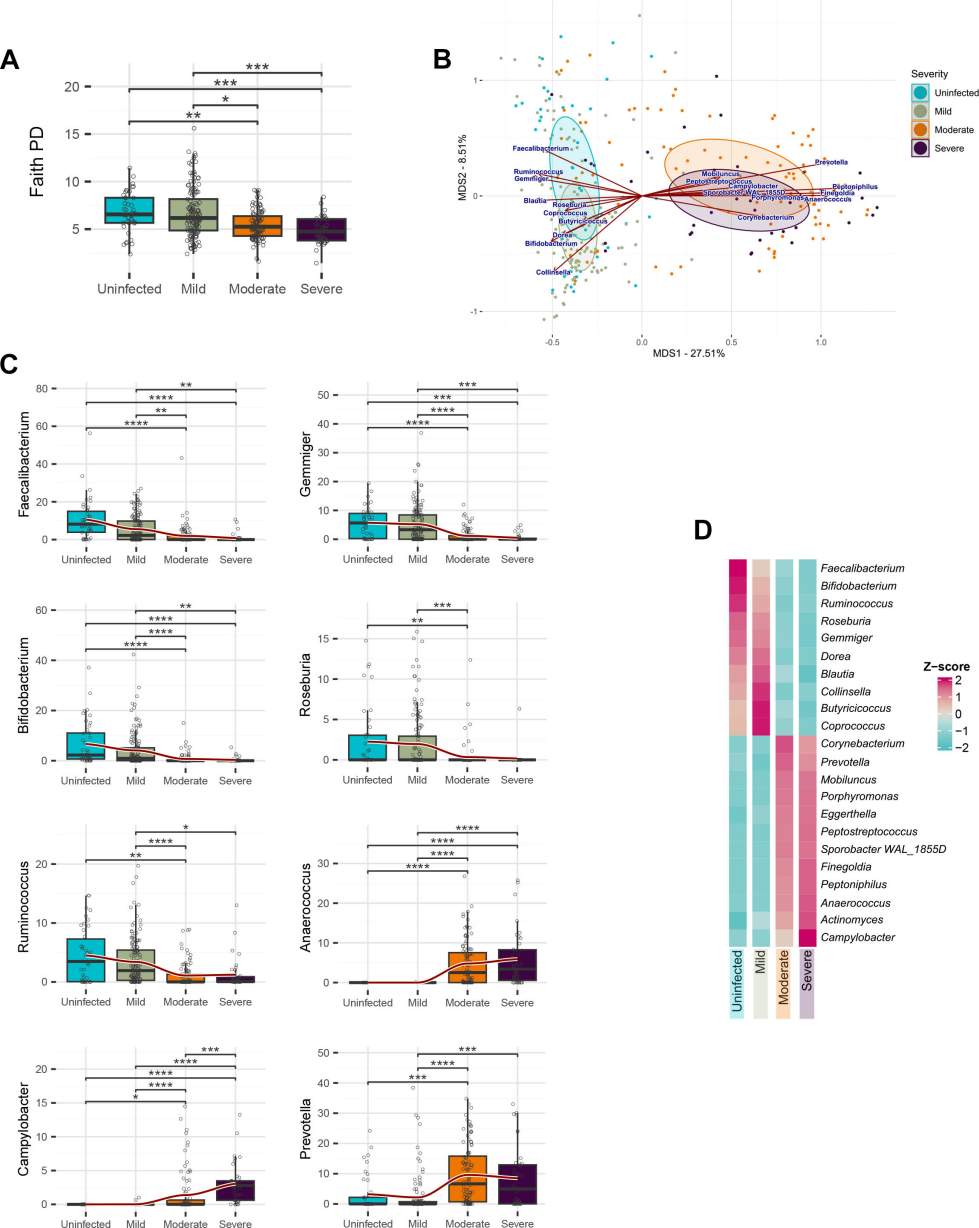
Severity-related differences in the gut microbiota of COVID-19 patients. (**A**) Alpha-diversity according to Faith’s phylogenetic diversity (Faith PD) in relation to COVID-19 severity classes (uninfected, mild, moderate, severe). (**B**) Principal coordinate analysis of weighted UniFrac distances shows segregation between non-hospitalized (uninfected and mild) and hospitalized (moderate and severe) subjects (pairwise PERMANOVA through the Adonis function –followed by Bonferroni correction, *P* = 0.0006). Arrows indicate the contribution of the fitted compositional variables (genus-level relative abundances) to the ordination plot (envfit, *P* ≤ 0.001). (**C**) Main genus-level compositional differences between severity classes, shown as boxplots of the overall distribution and C. See also Fig. S3. (**D**) Z-score scaled distribution of the main differences between severity classes. Only genera showing significant differences according to Bonferroni-corrected Kruskal-Wallis testing are plotted. Significance bars indicate *P*-values of Bonferroni-corrected pairwise Wilcoxon tests: *, *P* < 0.05; **, *P* < 0.01; ***, *P* < 0.001; ****, *P* < 0.0001.

Alpha-diversity varied significantly between the COVID-19 severity classes, according to Faith’s phylogenetic diversity (Wilcoxon pairwise tests with Bonferroni correction, *P* < 0.05) (Fig. 2A). In particular, it decreased with increasing severity, although no differences were observed between uninfected and mild groups, or between moderate and severe groups.

Beta diversity analysis, conducted using weighted UniFrac distances, revealed a marked contrast between the microbial configurations of the non-hospitalized severity groups and those of the moderate and severe inpatients (pairwise permutational multivariate analysis of variance [PERMANOVA] with Bonferroni correction, *P* = 0.0006) (Fig. 2B). Fitting the genus-level compositional data on the principal coordinate analysis (PCoA), we detected significant associations (envfit, *P* ≤ 0.001) between disease severity and several genera.

To seek for differences in the relative abundance of gut microbial components between severity classes, we used pairwise Wilcoxon tests, followed by Bonferroni correction for multiple comparisons. Several significant (*P* < 0.05) differences were found at the phylum (Fig. S1), family (Fig. S2) and genus level (Fig. 2C; Fig. S3). The leading differences between groups were attributable to the genera *Faecalibacterium*, *Bifidobacterium*, *Ruminococcus*, *Roseburia*, *Gemmiger*, *Dorea*, *Blautia*, *Collinsella*, *Butyricicoccus,* and *Coprococcus*, whose relative abundance was remarkably lower in the gut microbiota of hospitalized patients, and the potential enteropathogens *Anaerococcus* and *Campylobacter* as well as the opportunistic genera *Peptoniphilus* and *Finegoldia* that showed a significant blooming in the moderate and severe groups. Hospitalized patients showed a significant increase in the relative abundance of *Corynebacterium, Porphyromonas,* and *Prevotella* as well. Interestingly, *Campylobacter* was the only taxon that discriminated between moderate and severe inpatients, being overrepresented in the latter. On the other hand, no taxa were differentially represented between uninfected and mild subjects. Overall, the compositional analysis revealed severity-related signatures of the gut microbiota, with specific genera characteristic of no to mild infection and others characteristic of moderate to severe cases (Fig. 2D).

### Gut microbial networks related to COVID-19 severity

To complement the diversity and compositional insights with a more general overview of microbial interaction patterns, we performed a differential networking analysis at the genus level using SPRING (18). This tool was chosen because of its reported ability to take into account the peculiarities of microbiota amplicon data, i.e., being compositional and sparse.

**Fig 3:**
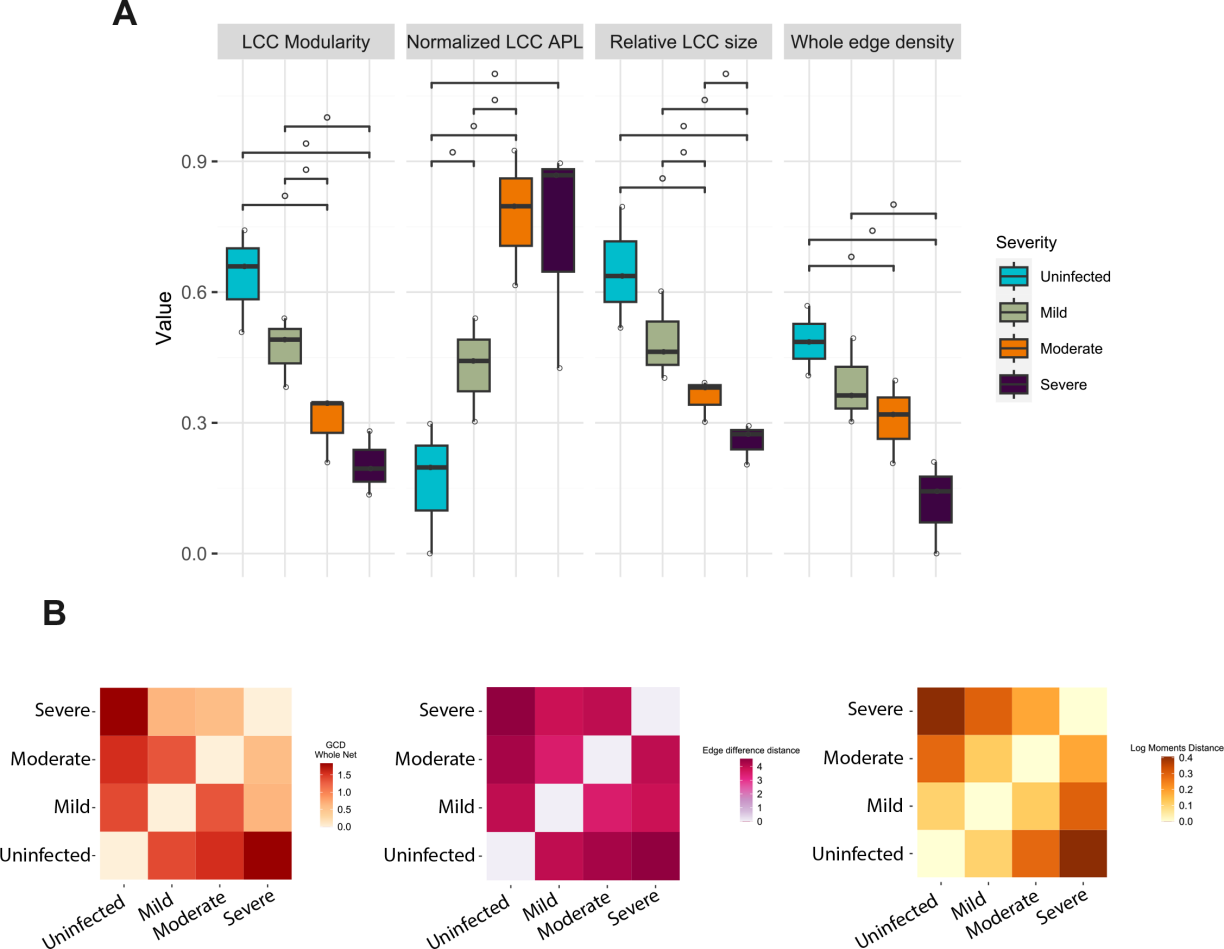
Local network properties differentiate the gut microbial community in COVID-19 patients with different symptom severity. (**A**) Topological parameters obtained from differential networking analysis conducted for all pairwise combinations of severity classes (uninfected, mild, moderate, severe), focusing on the LCC and whole network levels. APL, average path length. Significance bars indicate *P*-values of pairwise Wilcoxon tests followed by false discovery rate (FDR) correction: °, FDR < 0.1 (considered as a trend). (**B**) Pairwise distances between networks computed according to graphlet correlation distance (GCD; left), edge difference (center), and log-moments distance (right).

First, one network was reconstructed for each severity group for each pairwise comparison with all other severity classes (*n* = 3 networks computed for each group). Topological details concerning the largest connected component (LCC) and whole edge density were calculated for each network (Fig. 3A). To evaluate network topology, we calculated LCC modularity, LCC normalized average path length, LCC relative size, and whole network edge density (refer to Box 1 for the definition of such parameters).

Box 1. **Definition of network analysis terms used in this paper**

**Largest connected component (LCC)**: The subgraph of a network containing the largest group of nodes (in this case genera) in which there is a path between any two nodes. By focusing on the LCC, it is possible to evaluate the spread of information (in this case, the relationships between genera), as well as the robustness of the network to so-called “failures” (e.g., the loss of a microbial component).

**Modularity**: The degree to which a network is split into distinct, non-overlapping modules, whose nodes are more densely connected to each other than to the rest of the network. Low modularity values indicate that the nodes in the network do not form tight communities, so that an external pressure acting on specific nodes (causing the reduction or loss of microbial components) is more likely to spread to the other ones in the network, adding extra instability to the microbial community. On the other hand, high modularity—especially in the LCC subgraph—indicates that if one node fails, the failure is less likely to spread to other nodes, as the affected nodes would mostly be confined within the same module. This measure can be computed for the whole network as well as for subgraphs (e.g., the LCC only).

**Average path length (APL)**: A measure of the distance between nodes in the network, providing an estimate of the efficiency of communications between nodes. A reduced APL indicates that nodes are more closely connected and that the interconnections are stronger. This shall not be conflated with modularity, as a low APL does not necessarily imply that the network is less modular, as nodes can still be well organized into distinct modules. This measure can be computed for the whole network as well as for subgraphs (e.g., the LCC only).

**Relative LCC size**: Refers to the proportion of nodes included in the LCC compared to all the nodes constituting the network. A larger relative LCC size indicates that a greater proportion of the network is highly connected, suggesting the presence of stronger relationships between members, potentially leading to improved resistance to stressors and resilience.

**Whole network edge density**: A measure of network connectivity. It provides a way of comparing networks regardless of their size or number of nodes, which is very useful in the case of differential network analysis. A low edge density means that most nodes are connected to only a few other nodes, suggesting that there are poor relationships between the microbial components, potentially resulting in an unbalanced ecosystem.

As disease severity escalated, the topological structure of the network LCC showed progressively lower modularity, higher normalized average path length, and lower relative size. In addition, the whole network edge density was found to decrease in a severity-related manner.

Next, several mathematical measures were calculated to quantify the similarity between network pairs. Specifically, we used the graphlet correlation distance, the edge difference distance, and the log-moments distance (Fig. 3B). All three distances showed a progressive increase in differences in network topology between severity classes with increasing severity, confirming that the overall configuration of microbial ecosystems is linked to COVID-19 severity.

**Fig 4:**
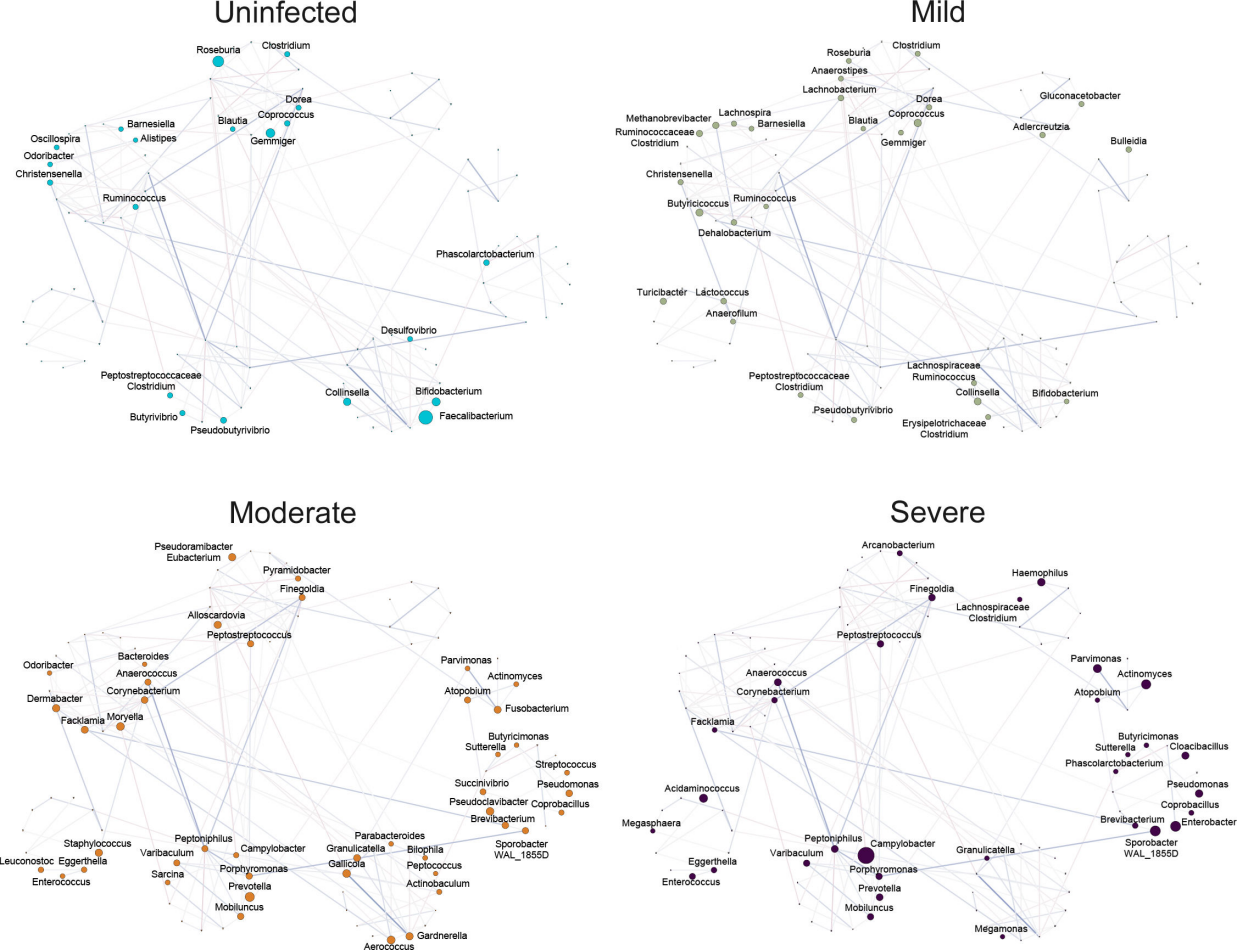
Global networking detects COVID-19 severity-related gut microbiota modules. A single network was constructed from all samples and modules were detected using a spin glass model. Modules are highlighted with colored circles encompassing the enclosed nodes. For each severity class (uninfected, mild, moderate, severe), the network was plotted setting the node size proportional to the overabundance of that taxon in the corresponding group. Only nodes with an overabundance of at least 1.35 were plotted and labeled. Edge width was set proportional to the adjacency values obtained from NetCoMi netConstruct and colors indicate positive (blue) and negative (red) interactions.

Finally, to graphically blend the community properties obtained from the global topological analysis with the microbial composition data, we moved from a differential local networking approach to a global networking approach, reconstructing a single network of interactions for the entire data set. Using a spin glass model and simulated annealing model, we identified eight distinct modules in this network and evaluated the overabundance of nodes in each severity group (Fig. 4). This analysis showed a gradual change in the way the modules were populated in the severity groups, with a progressive blooming of the aforementioned genera (e.g., *Anaerococcus, Campylobacter, Peptoniphilus, Finegoldia, Porphyromonas, Corynebacterium* and *Prevotella*) with increasing severity, accounting for the presence of clear changes in several network parameters describing ecological relationships likely to occur in the microbiota.

### Predictive potential of gut microbiota in COVID-19 patient triage

**Fig 5:**
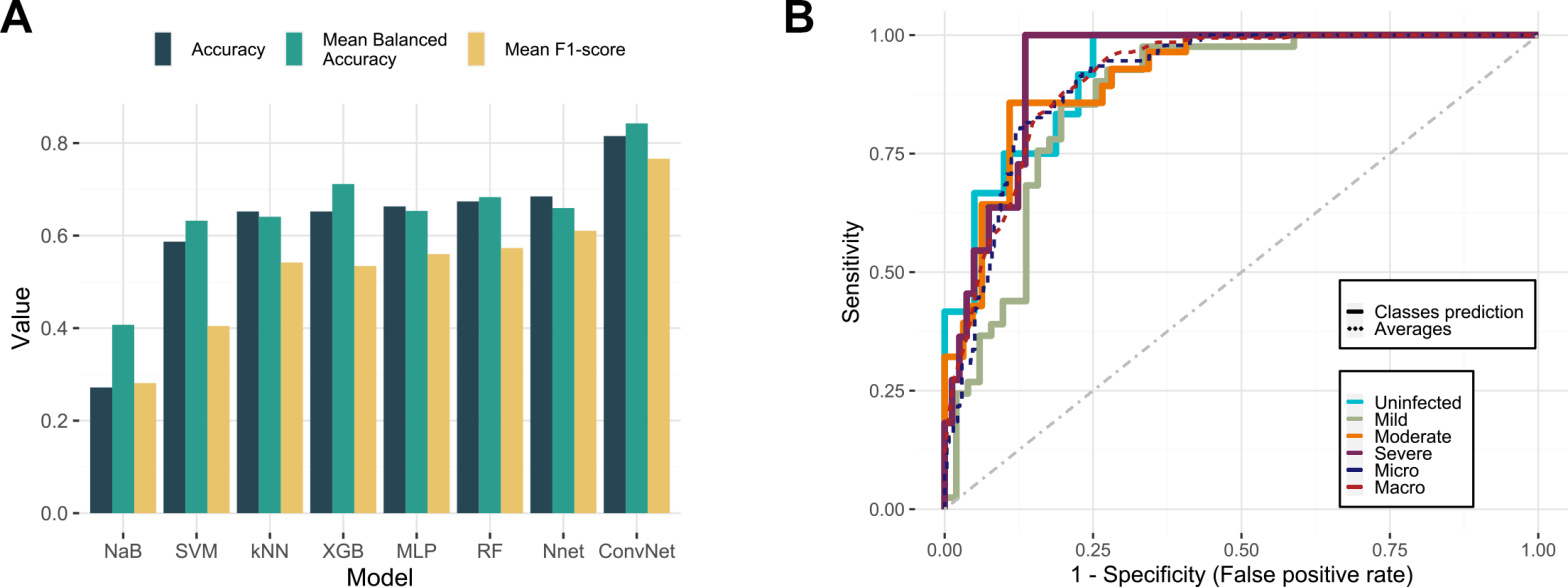
Gut microbiota as a predictor of COVID-19 severity. (**A**) Summary of the performance of the several classifiers tested over the test set. The ConvNet with hyperparameter tuning achieved the best results in terms of accuracy, mean balanced accuracy, and mean F1-score. (**B**) Multi-class ROC sensitivity/false-positive rate curves for the ConvNet model. Continuous lines represent the prediction for the severity classes (uninfected, mild, moderate, severe), while dashed lines represent the macro- and micro-averages. NaB, naïve Bayes; Nnet, neural net in caret.

Considering the early signatures detected in the gut microbiota in the 5- to 10-day window after symptom onset, we hypothesized that the gut microbiota might be one of the biomarkers that could aid in the decision-making process and triage of patients during SARS-CoV-2 infection, especially in this critical timeframe when the disease could progress toward moderate and severe manifestations. To test this assumption, we trained and tested multiple machine-learning classifier algorithms to predict disease severity based on the genus-level microbial composition of patients’ stools. Specifically, we implemented eight different machine-learning classifier algorithms and evaluated their performance via accuracy, mean balanced accuracy, mean F1-score, and receiver operating characteristic (ROC) sensitivity/false-positive rate curves. The machine-learning classifiers were (i) a *k*-nearest neighbor (k-NN) classifier, (ii) a random forest (RF) classifier, (iii) a neural network using the caret R package, (iv) a naïve Bayes classifier, (v) a support vector machine (SVM) working in a one-vs-all manner implementing the e1071 R package, (vi) a XGBoost (XGB) classifier using the xgboost package in R, (vii) a multi-layer perceptron (MLP); and a (viii) Convolutional Neural Network (ConvNet) using the scikit-learn, keras, and tensorflow python libraries (see Materials and Methods). The last showed the best results compared to the other classifiers in terms of accuracy (0.815), mean balanced accuracy (0.842), F1-score (0.766) (Fig. 5A), and area under the curve values (micro = 0.905, macro = 0.909) (Fig. 5B).

Our proof-of-concept classifier achieved acceptable results, demonstrating that gut microbiota has the potential to be considered as a biomarker at the early stages of infection, potentially facilitating patient triage and appropriate treatment.

### Evaluation of potential selection bias and validation of model performance on external data

Our data may be subjected to potential selection bias, since the geographical origin of the samples is also associated to the classification of disease severity in our cohorts. Hospitalized subjects originate mostly from the Verona cohort in Italy, while non-hospitalized individuals belong to the COVID-HOME cohort in Groningen, The Netherlands. This potential selection bias could affect the interpretation of the results as demographic, age, body mass index (BMI), and biological sex differences between the two cohorts may be major confounding factors of the effective relationship between microbiota and disease severity. In an attempt to investigate such potential bias and validate our results, we firstly compared the samples within each of the two cohorts that fell under the same severity classes, that is intra-mild and intra-moderate groups (Fig. S4A through C). Understanding the precise characteristics of these two classes is pivotal, as they represent the interface between non-hospitalized and hospitalized subjects. Additionally, they are the only overlap between the two cohorts in terms of severity classes. We found no significant cohort-driven differences in the intra-severity comparison of alpha- and beta-diversities, as well as in the overall average composition at the genus level. Nevertheless, it is important to acknowledge the small sample size considered for these comparisons; only four mild individuals were included from the Verona cohort (against 135 from Groningen), and only seven moderate participants originated from the Groningen cohort (vs 89 from Verona). To further evaluate the robustness of our findings, we gathered human gut microbiota 16S rRNA data from external data sets (15, 19) that were profiled following our same pipeline (Table S1 for external cohort characteristics and Table S2 for the respective sequence accessions). The external data set used comprised 223 human gut microbiota samples obtained from COVID-19 patients during the acute phase of the disease, stratified according to the WHO clinical progression scale. At a first glance, the external data showed similar distribution to our data, according to beta-diversity stratifying for disease severity (Fig. S4D). We then deployed our ConvNet model on the microbiota composition of these samples to test predictions on disease severity. The resulting confusion matrix (Table S3) demonstrates that even in cases of incorrect predictions, the classifier maintained a relatively close alignment with the actual severity class. The ROC curves (Fig. S4E) show an acceptable balance between sensitivity and false-positive rate, with a micro-average of 0.837 and a macro-average of 0.838. Evaluating the classification goodness, we achieved an overall accuracy of 0.726, mean balanced accuracy of 0.808, and an F1-score of 0.736 (Fig. S4F). These statistics indicate a robust generalization and prediction capability of our model over external data, suggesting a strong link to COVID-19 severity classes and ruling out an association to cohort demographics.

## DISCUSSION

In this work, we described the relationship between gut microbiota and COVID-19 severity, considering the microbial ecosystem during the first week post-symptom onset, the corresponding stage of disease progression in non-hospitalized subjects, and patient outcome. Our cohort, with a total of 315 fecal samples from as many individuals, represents one of the largest COVID-19 cohorts profiled using 16S rRNA gut microbiota investigation technique to date and is part of the ORCHESTRA project, one of the largest EU efforts to gather knowledge from COVID-19 pandemics. We made use of both compositional analysis and networking approaches to disentangle the putative contribution of microbial components to COVID-19 severity.

In particular, we detected a severity-related dysbiotic configuration in the gut microbiota with reduced ecosystem diversity, as expected according to previous studies (20, 21). Similarly expected, as COVID-19 severity increased, several typically healthy-associated taxa such as *Faecalibacterium*, *Bifidobacterium*, *Ruminococcus*, *Roseburia*, *Dorea*, *Blautia*, *Butyricicoccus,* and *Coprococcus* were found in reduced amounts. These genera are known to produce short-chain fatty acids, which are immunomodulatory molecules that exert anti-inflammatory effects on the host, taming the side effects of the immune response (22–26). On the other hand, as previously reported (14, 27), an increase in potential enteropathogens was detected in COVID-19 inpatients, with blooming colonization by *Campylobacter*, *Anaerococcus*, *Peptoniphilus*, *Finegoldia*, *Porphyromonas*, *Corynebacterium,* and *Prevotella*.

*Campylobacter* is a pathogenic genus causing diarrhea, abdominal pain, fever, and sometimes vomiting. The proliferation of *Campylobacter* can disrupt the microbiota balance and trigger an immune response in the gut, leading to inflammation (28), possibly contributing to or exacerbating COVID-19 symptoms in hospitalized patients. Increases in the relative abundance of *Campylobacter* in both the upper respiratory tract (29) and the gut (30) have already been reported in severe COVID-19 patients. Similarly, the opportunistic pathogens *Anaerococcus* and *Peptoniphilus* have been found to be enriched in COVID-19 patients (31), especially those prone to colonization by multi-drug-resistant bacteria (32). Less information is available on *Finegoldia*, *Porphyromonas,* and *Corynebacterium*, but all of them have been associated with COVID-19 as well (33). Finally, *Prevotella* is a fiber fermenter often considered as a marker of gut eubiosis given its pivotal contribution to polysaccharide breakdown (34). However, this genus has a high genetic diversity, and several articles have reported an increase of its abundance in association with viral infections such as SARS-CoV-2, human immunodeficiency virus, papillomavirus, and *Herperviridae* (35). Our results confirm previous findings and pose new questions for future studies. In particular, as certain *Prevotella* spp. can produce certain proteins that can promote viral infection (36), additional deep shotgun metagenomics studies will be required to investigate the fostering mechanisms of *Prevotella*-mediated inflammation. Overall, the reduction of health-associated anti-inflammatory genera and the enrichment in potential enteropathogens may lead to immune dysfunction and inflammation, which may impact the course of an ongoing viral infection as COVID-19.

A few other studies have implemented networking approaches to evaluate the gut microbiota in COVID-19 (15, 37, 38), but the cohorts considered were smaller or lacked an even distribution of all disease severity classes as performed in our work. Our networking analysis operated locally, i.e., inferring networks of interaction at the level of individual severity classes allowed us to shed light over the properties of the microbial community. Specifically, we detected alterations in several parameters related to community plasticity and overall stability (namely reduced modularity, LCC size, and whole network edge density, and increased average path length) going hand in hand with the increase in severity. Moderate and severe groups showed networks with poor relationships between members, with a configuration more prone to the propagation of potential failures due to environmental stress, with a plausible reduction in the ability to maintain positive crosstalk with the host (39, 40). Considering that both compositional and networking analyses highlighted how different the gut microbiota configuration is depending on disease severity, we then moved to a global networking approach, where a single network was constructed for all samples to evaluate all microbial interactions possibly taking place in our cohort, detecting modules of taxa closely interacting with each other and observing how the different severity classes populated such modules in terms of overabundance. The analysis revealed that the blooming of certain taxa in higher severity classes established a completely different pattern of interactions, suggesting an actual reshaping of the microbial configuration. A previous research article exploiting networking approaches applied to the gut microbiota in COVID-19 highlighted the relevance of the interactome of a microbe (i.e., the properties of its connections to other members of the network, determining network topology) in determining the clinical status of COVID-19, especially disease severity (38). In this regard, our results confirm the link between COVID-19 severity and not only the relative abundance of several gut microbiota components, but also the properties of the ecological links among its members. Despite the implementation of a different networking approach, our results are overall consistent with those reported by Reinold et al. (15), which highlight a reduction in the connectivity of the network structure representing the gut microbiota, with the loss especially affecting genera such as *Faecalibacterium* and *Roseburia*, as we found.

Given such marked differences observed in relation to severity classes, the relatively large sample size of our study, and considering the potential life-saving effect of early discrimination between severity classes, for the prompt tagging of severe cases at first hospital admission, we trained, optimized, validated, and tested a machine-learning multi-class classifier exploring a wide range of model logics and architectures. Benchmarking the different classifiers on the test set, we achieved an accuracy of 81.5%, a mean balanced accuracy of 84.2%, and an F1-score of 76.6%. The coverage of the area under the multi-ROC curves obtained for the different classes ranged from 90.5% to 90.9% (micro- to macro-averages). These values are in line with those previously reported for determining an efficient prediction model in different microbiome-based multi-class classification tasks (41–43). Although our model is far from being used in clinical practice, not least because of the processing time required for compositional analysis, our results serve as a proof of concept that should encourage the enrollment of even larger cohorts for gut microbiota analysis and bolster the use of gut microbiota as a potential biomarker (and eventual target for microbiota-modulating strategies) to be considered in support of clinical decision-making.

The main limitations of our study are as follows: (i) the timing of fecal sampling, which was not strictly standardized but varied within a period of 5–10 days from the onset of the first symptoms; (ii) the sample size, considering the usual amount of data required to derive large-scale robust machine-learning models that can be efficiently deployed in clinical practice; (iii) the mixed use of rectal swabs and fecal samples, although several studies have reported that rectal swabs are reliable alternatives to whole stool collection and provide comparable results, especially when, as in this case, the samples are processed in the same laboratory (44–46); and (iv) the lack of consideration of host metadata, such as age, biological sex, BMI, therapy, etc., which may have biased our results. Finally, we were not able to distinguish all severity classes based on gut microbiota profiles using statistical approaches, since uninfected and mild subjects had similar patterns, as did moderate and severe patients. However, by leveraging machine-learning approaches that can better disentangle non-linear relationships between features, we were able to successfully predict all severity classes, with satisfactory accuracy and precision. Furthermore, our work relies on standardized definitions of COVID-19 severity and features a relatively large sample size, considering the nature of the samples.

We attempted to address some of such limitations exploring the possible cohort-related biases in the intra-mild and intra-moderate severity classes between the two cohorts. Despite modest sample sizes, no significant cohort-driven differences emerged in alpha- and beta-diversities or genus-level composition. To bolster our findings, we integrated external data sets, revealing our ConvNet model’s robust generalization and confirming prediction capabilities. The classifier, even in misclassifications, closely tracked the true severity class, with ROC curves showing a balanced trade-off. The model’s accuracy on external data was quite similar to the performance detected on the internal test set, highlighting the model’s reliability over diverse data, strengthening the evidence for a substantial link between gut microbiota and COVID-19 severity, transcending cohort-specific characteristics. Nevertheless, these results underscore a relationship between the GM and COVID-19 severity that falls short of being causative for ARDS. The microbial signatures identified in association with disease severity are likely contributors to the intricate host mechanisms influencing various outcomes in SARS-CoV-2 viral infection.

While our results underscore a profound rewiring in gut microbiota composition associated with COVID-19 severity, it is essential to clarify that our findings demonstrate correlation rather than causation. The observed changes in bacterial taxa suggest a potential link to disease severity, influencing ecological interactions and community properties. These alterations could exacerbate host health issues, intensify inflammatory responses, and potentially contribute to complications. Our evidence strengthens and enriches what has been previously reported, bolstering interest in the future use of gut microbiota to predict patient outcomes and develop targeted therapies in conjunction with COVID-19 treatments. To this end, microbiota-modulating targeted therapies (e.g., the use of probiotics, prebiotics, phage therapy, engineered bacteria, or fecal microbiota transplantation) could be considered as a means to shift the configuration to a healthier state early in the disease course. While these interventions may hold promise, their impact on patient outcomes requires further investigation, and their potential benefits should be considered within the context of ongoing research and clinical trials.

## MATERIALS AND METHODS

### Subject enrollment and fecal sampling

Subjects included in this work were enrolled during the first, second, and third waves of the SARS-CoV-2 pandemic in the context of the EU project ORCHESTRA (Connecting European Cohorts to Increase Common and Effective Response to SARS-CoV-2 Pandemic) (https://orchestra-cohort.eu/). The recruiting centers involved in this study were (i) the COVID-HOME cohort (17) of the Department of Medical Microbiology and Infection Prevention, University Medical Center Groningen, Groningen, The Netherlands, initially funded by Netherlands Organisation for Health Research and Development (ZonMw) and later by the ORCHESTRA project, and (ii) the Infectious Diseases Unit, Department of Diagnostics and Public Health, University of Verona, Verona, Italy. Patients enrolled in Verona (Italy) were sampled using rectal swabs at hospital admission after laboratory-confirmed diagnosis of SARS-CoV-2 via nose swab PCR. The COVID-HOME cohort enrolled nose swab-confirmed SARS-CoV-2 subjects who spent their positivity at home, as well as all their housemates, including healthy nose PCR-tested uninfected subjects, who were asked to provide a stool sample. All samples were collected between day 5 and day 10 of symptom onset and stored at −80°C until further processing. A total of 315 subjects were included in this work (see Fig. 1 for sample distribution across recruiting centers, severity classes [i.e., uninfected, mild, moderate and severe], and sampling timing). All study participants provided written informed consent before participating in the research (17).

The validation cohort has been collected from two publicly available studies (15, 19), including a total of 223 samples covering all the severity classes, balanced in sex and age (validation cohort details in Table S1).

### Microbial DNA extraction, library preparation, and 16S rRNA amplicon sequencing

All samples were processed under biosafety level 3 (at the Centro di riferimento regionale per le emergenze microbiologiche, St. Orsola Hospital, Bologna, Italy). Microbial DNA was extracted using an improved version of the repeated bead-beating plus column method (47, 48). The V3–V4 hypervariable regions of the 16S rRNA gene were then amplified using the 341F and 785R primers (49) for the library preparation step. The final libraries, indexed and purified, were sequenced on an Illumina MiSeq platform using a 2 × 250 bp paired-end protocol according to the manufacturer’s instructions. The 16S rRNA amplicon sequencing of the samples resulted in 65,608 ± 1,361 (average ± SEM) raw reads per sample. Sequencing raw reads were deposited in the National Center for Biotechnology Information Sequence Read Archive (NCBI SRA; BioProject ID PRJNA1031953). Sequences from the validation cohort were gathered from the NCBI SRA under publicly available BioProject codes PRJNA684070 and PRJNA747262, showing an average of 18,6011 (±2,630; SEM) reads per sample. The list of the 223 samples used to deploy our model can be consulted in Table S2.

### Gut microbiota compositional analysis

Raw sequences were processed using a combined pipeline of PANDASeq (50) and QIIME 2 2023.2 (51). Quality-, chimera-, and length-filtered reads were grouped into amplicon sequence variants (ASVs) using DADA2 (52). Taxonomic assignment was performed using a hybrid method consisting of VSEARCH (53) and a naïve Bayes classifier trained on the Greengenes 13.8 database according to the QIIME q2-feature-classifier plugin indications (54). To address variations in sequencing depth among samples, taxonomic counts tables were rarefied at 1,091 counts for our study cohort and at 1,492 counts for the external deployment data set. Diversity was calculated using the qiime diversity core-metrics-phylogenetic plugin. Weighted UniFrac beta-diversity distances were used as input for PCoA using the “vegan” R package (55). Data separation was tested with a pairwise permutation test with pseudo-F ratio using the pairwiseAdonis R package (56) and corrected using the Bonferroni method. The bacterial genera contributing significantly to the ordination distribution were identified using the “envfit” function from “vegan.” PCoA plot was generated using the ggplot2 and shadowtext (57) R packages. Differences in alpha-diversity and relative taxon abundances were tested using Kruskal-Wallis testing followed by *post hoc* pairwise Wilcoxon rank-sum tests, followed by correction for multiple comparisons using the Bonferroni method. A corrected *P*-value <0.05 was considered significant. Boxplots were generated using the ggplot2 (58) R package and significance was plotted using the ggsignif R package (59). Smoothed conditional means on the genus-level relative abundance plots were generated using the “geom_smooth” function from ggplot2 using the “loess” method. The genus-level heatmap was plotted using ggplot2 and genera were ordered according to significant Kruskal-Wallis Bonferroni-corrected *P*-values (*P* < 0.05).

### Differential and global network construction and evaluation

Network construction and differential networking analysis were performed using the NetCoMi R package (60) from genus-level ASV count data, considering only taxa with significant variation across severity groups according to Kruskal-Wallis Bonferroni-corrected *P*-values. Differential networks were constructed based on SPRING (18) association via the “netConstruct” function of the NetCoMi package. The parameter set included adaptive Benjamini-Hochberg multiple testing adjustment, default preprocessing, and setting both “normMethod” and “zeroMethod” to “none” as normalization and zero handling are performed internally by SPRING. No sparsification was performed, as SPRING already returns a sparse network where no additional sparsification step is necessary. The “signed” method was used to transform associations into dissimilarities, which were passed to the “dissFunc” parameter. Differential network analysis was conducted using the “netAnalyze” function from NetCoMi, setting the clustering method to “cluster_louvain” with weighted clustering coefficients, and computing normalized degree, betweenness, closeness, and eigenvector centrality measurements only for the LCC of the network. Finally, pairwise network comparisons were performed using the “netCompare” function from NetCoMi, with permutation testing, adaptive Benjamin-Hochberg multiple comparison correction, and a quantile threshold of 0.80 for Jaccard’s index calculation. The summaries of such pairwise comparisons were used to gather data on network topology. Gathered values were tested for significant differences using Wilcoxon pairwise tests followed by Benjamini-Hochberg false discovery rate correction.

An additional network topology comparison was performed using the NetworkDistance R package (61). The association matrices obtained from the reconstructed NetCoMi networks were passed to the “nd.edd” and “nd.moments” functions of the NetworkDistance package to return the edge difference distance and log-moments distance matrices, respectively, comparing all the networks. Heatmaps were plotted using the ggplot2 R package.

For global network construction, the “netConstruct” NetCoMi function was used as described above, with the group = NULL formula to compute a single network. The edge list table was used to compute modules using the “cluster_spinglass” function from the igraph R package (62), setting the “random” update rule, the “neg” implementation, and both “gamma” and “gamma.minus” parameters to 0.8. Global networks were plotted using Cytoscape software (63), displaying the modules according to the spin glass group membership. The overabundance of each taxon in each group was computed as the mean relative abundance in that group divided by the mean relative abundance in the entire data set. Only nodes and node labels with an overabundance of at least 1.35 were plotted, and node size was set proportional to the overabundance value in the corresponding severity group. Edges were labeled according to the association value and the line width was set proportional to the adjacency value reported by the “netConstruct” function in the edge list table.

### Machine-learning classifier training and performance testing

To test whether the genus-level composition of the gut microbiota 5–10 days after symptom onset (concurrent with usual hospitalization occurrence) could predict COVID-19 severity, we trained, hyper-tuned, and tested eight different classifiers. The data set was prepared by retaining only the 22 most relevant taxa detected as significantly different by Kruskal-Wallis testing (Fig. 2D), and then split into training and test sets in a 70–30 fashion. Each model was trained on the training set—with a known severity class assigned to each sample—and then challenged against the test set to make predictions.

Most of the models were constructed using the caret R package (64). The pre-processing function used for caret models included removal of near zero-variance predictors, data centering, and scaling (passing the “center” “scale,” and “nzv” methods to the “preProcess” function). Cross-validation was performed using the RepeatedCV method, with eight complete sets of folds, with 30 resampling iterations each, and the “random” grid tuning method. The summary metric used to select the optimal model was set to “accuracy.” The random forest model was trained growing 1,500 trees. The resulting model was used to cast severity class probabilities against the test set, then the resulting confusion matrix was used to assess model performance via the “multiClassSummary” function from caret. Accuracy, mean balanced accuracy, and mean F1-score were gathered for each model and used to compare the models. The kNN was trained similarly using caret, adopting the same pre-processing and cross-validation parameters. In addition, the “tuneLength” parameter was set to 50. We trained a neural network with caret using the “nnet” method, with the same pre-processing and cross-validation parameters as before, reducing the “tuneLength” value to 20. We then trained an SVM in a one-vs-all configuration using the “svm” function from the e1071 R package (65). We set a cost of 30, a tolerance equal to 0.0001, and used a linear kernel for the C-classification training of the SVM. Model performance was assessed in the same way as before, i.e., with the “multiClassSummary” function. The e1071 package was used to train the naïve Bayes classifier as well, with the “naiveBayes” function with default parameters. Performance was tested likewise. The XGBoost classifier was trained using the xgboost R package (66). Briefly, the training and test sets were formatted accordingly with the “xgb.DMatrix” functions; we set the training step to 500 rounds, 10 cross-validation folds, with the “multi:softprob” objective and the “mlogloss” evaluation metric. The model was trained with the “xgb.train” function and tested on the test set to produce a confusion matrix. Model performance details were gathered as previously. We then constructed an MLP and a ConvNet using the scikit-learn (67) 1.2.1, tensorflow (68) 2.11.0, and keras (69) 2.11.0 Python modules. Using the GridSearchCV function, we tested different estimators, number of layers, and neurons in each layer, with or without dropout layers at variable dropout rates, different initializers, activation functions for each layer and training epochs, and kernel sizes for the ConvNet, for a total of 10 cross-validations for each combination.

In detail, for MLP, we tested a model with as little as one input and one output layer, with as low as eight input neurons and four output neurons, up to four layers with as many dropout layers between each, testing the combination of 8/16/26/32 neurons in each and 0.0 to 0.5 (increasing by 0.1) dropout rates. In addition, we permuted these tests with all possible combinations to test the Adam, RMSProp, and SGD optimizers, the Glorot uniform, He normal and Lecun uniform initializers, and the ReLU, sigmoid, and tanh activation functions. All these combinations were tested for 100 to 500 epochs, with 100 jump increments. The best hyperparameters obtained (three layers: 22 starting neurons with Lecun uniform kernel initializer and sigmoid activation, followed by two blocks consisting of an eight-neuron layer with the same activation function and a 0.1 dropout layer, to ultimately reach the four-neuron output layer with ReLU activation and RMSProp optimizer) were used to train the model using the binary cross-entropy loss evaluation function and observing the accuracy metric during the process. Once the model was trained, we ran a prediction over the test set, generated a confusion matrix, and gathered model performance details as described above.

For the ConvNet, on the other hand, we tested for a sequential model, consisting of multiple layers of convoluted neurons, followed by a pooling layer. Subsequently, a dropout layer and a flatten layer were added, followed by a single dense layer with dropout, to ultimately reach the output layer. We tested up to four nested convoluted/pooling layer combinations, with 8, 16, 22 neurons, (3,3), (5,5), (7,7) kernel sizes, ReLU, sigmoid, and SoftMax activation functions, and rendered the output with the same padding as the input shape. For the pooling layers, we always used the (1,1) pooling shape. The dropout layer was tested with dropout values ranging from 0.0 to 0.5 with 0.1 jump increments, and the neurons tested for the dense layer were 4, 8, 16. We tested three different optimizers (Adam, SGD, RMSprop), batch sizes of 32, 64, 128, and 256, and epochs from 100 to 500, with 100 increments. The loss function used during all permutations was categorical cross-entropy and we monitored the accuracy metric. The final model included three convoluted layers of 16 neurons, with sigmoid activation function, a 7 × 7 kernel, rendering an output with same padding as the input. No pooling layer was introduced, and the Conv2D output was flattened and fed to the four-neuron output layer, with sigmoid activation. The final model was compiled with categorical cross-entropy loss function, the RMSprop optimizer, and first evaluated using accuracy. Once trained, we performed prediction and performance testing as described above.

Parameters for model performance evaluation were plotted as bar plots using ggplot2. The multiROC R package (70) was used to generate ROC curve data for the multi-class classifiers, and the results were plotted using ggplot2.

The best model achieved was then deployed on external data to assess generalization capabilities. This means that such model was initially trained on the microbiota composition of the 315 samples collected from the Verona and Groningen cohorts and presented in this study. Subsequently, once trained, it was asked to predict the disease severity of the 223 external data samples, taking in input the relative abundance value of the 22 relevant taxa selected. Therefore, the external samples were entirely distinct from those used for training, allowing for an evaluation of the model’s generalizability to unseen data.

### Detection of SARS-CoV-2

COVID-19 diagnosis relied on RT-PCR analysis of nasopharyngeal swab samples, stool samples, and serological confirmation of SARS-CoV-2 antibody presence. The qRT-PCR specifically targeted the E-gene of SARS-CoV-2 as per the method by Corman et al. (71). RNA extraction from 190  µL of each sample was performed using the NucliSense EasyMag (bioMérieux, Lyon, France), and the Fast Virus 1-Step kit (Thermofisher Scientific) was utilized with 10 µL of extracted RNA, resulting in a total reaction volume of 25 µL. The PCR cycling conditions were conducted on an ABI 7500 (Life Technologies) with an initial step at 50°C for 15 minutes, followed by 45 cycles of 5 seconds at 95°C, 5 seconds at 50°C, and 45 seconds at 60°C. For antibody detection, the semi-quantitative Architect IgM and IgG enzyme-linked immunosorbent assays (ELISAs) were employed.

### Isolation and library preparation

Samples with Ct <30 were grouped based on Ct-values for downstream pooling compatibility. Viral RNA was extracted from positive SARS-CoV-2 samples, where Ct ranged from 12 to 30, using NucliSense EasyMag (bioMérieux) and stored at −20°C. A cDNA synthesis reaction was prepared using LunaScript RT supermix (5×), RNA samples, and nuclease-free water. The cDNA synthesis involved primer annealing at 25°C for 2 minutes, cDNA synthesis at 55°C for 20 minutes, heat inactivation at 95°C for 1 minute, and cooling down to 4°C. The cDNA was then prepared for the pre-PCR environment and proceeded with the RT-PCR protocol.

### Whole genome sequencing (WGS)

For further analysis, WGS was conducted using the EasySeq RC-PCR SARS-CoV-2 WGS kit (NimaGen BV, Nijmegen, The Netherlands). Two sets of primers were used for targeted amplification, generating specific SARS-CoV-2 products with Unique Dual Index and adapter included. The amplicon fragment size in the final library was approximately 435 bp. Sequencing was carried out on the Illumina MiSeq platform with a Mid Output Kit (2 × 250 cycles) (Illumina, San Diego, CA, USA), loading a final concentration of 9pM for the library pool.

### Consensus generation and clade assignment

Analysis of paired-end viral FASTQ files was performed using the CLC Genomics Workbench v21.0.4 (CLC, Qiagen, Hilden, Germany). Reads were trimmed (Qscore >30) and mapped randomly against SARS-CoV-2 Wuhan-Hu-1 (accession no: NC 045512.2) using affine gap cost. Subsequently, read mappings were locally realigned, primers were trimmed, and Indels and structural variants were calculated. The coverage threshold per position for consensus generation was set at 30×. Clade assignments for each consensus were determined using Nextclade (https://clades.nextstrain.org/).

## Data Availability

Sequences generated in this study are publicly deposited in the National Center for Biotechnology Information Sequence Read Archive (NCBI SRA), accession code: PRJNA1031953. The sequences comprising the validation cohort were obtained from the NCBI SRA, accessible through the publicly available BioProject codes PRJNA684070 and PRJNA747262. For detailed information on the 223 samples utilized for deploying our model, please refer to Table S2.

## SUPPLEMENTAL MATERIAL

Supplemental Figures msphere.00181-24-s0001.pdf Figures S1 to S4.

Supplemental Tables msphere.00181-24-s0002.xlsx Tables S1 to S3.

ASM does not own the copyrights to Supplemental Material that may be linked to, or accessed through, an article. The authors have granted ASM a non-exclusive, world-wide license to publish the Supplemental Material files. Please contact the corresponding author directly for reuse.
