# Supplementary material for: The gut microbiota as an early predictor of COVID-19 severity
Source: mSphere. 2024 Sep 19;9(10):e00181-24. doi: 10.1128/msphere.00181-24 (PMC11540175; doi:10.1128/msphere.00181-24)
Supplement: Supplemental Figures — Figures S1 to S4. [file msphere.00181-24-s0001.pdf]

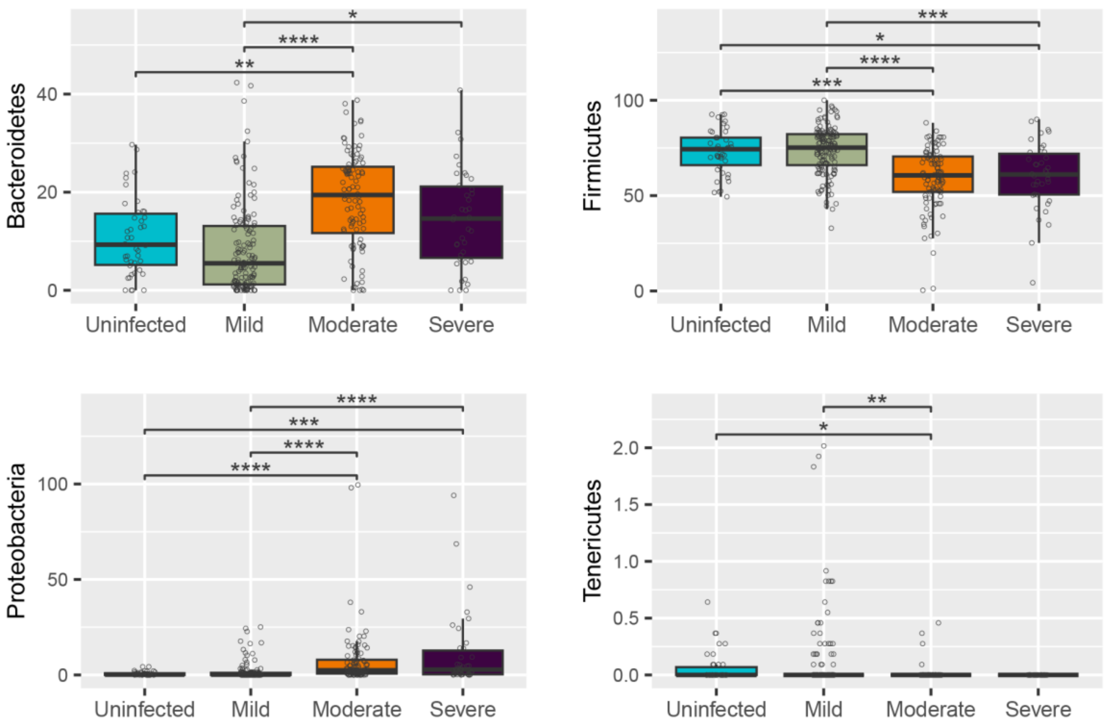

Supplementary Figure 1. Phylum-level differences in gut microbiota related to COVID-19 severity.

Boxplots showing the relative abundance distribution of phyla differentially represented between COVID-19 severity classes (uninfected, mild, moderate, severe). Significance bars indicate *p* values of Bonferroni-corrected pairwise Wilcoxon tests: *, $p < 0.05$; **, $p < 0.01$; ***, $p < 0.001$; ****, $p < 0.0001$.

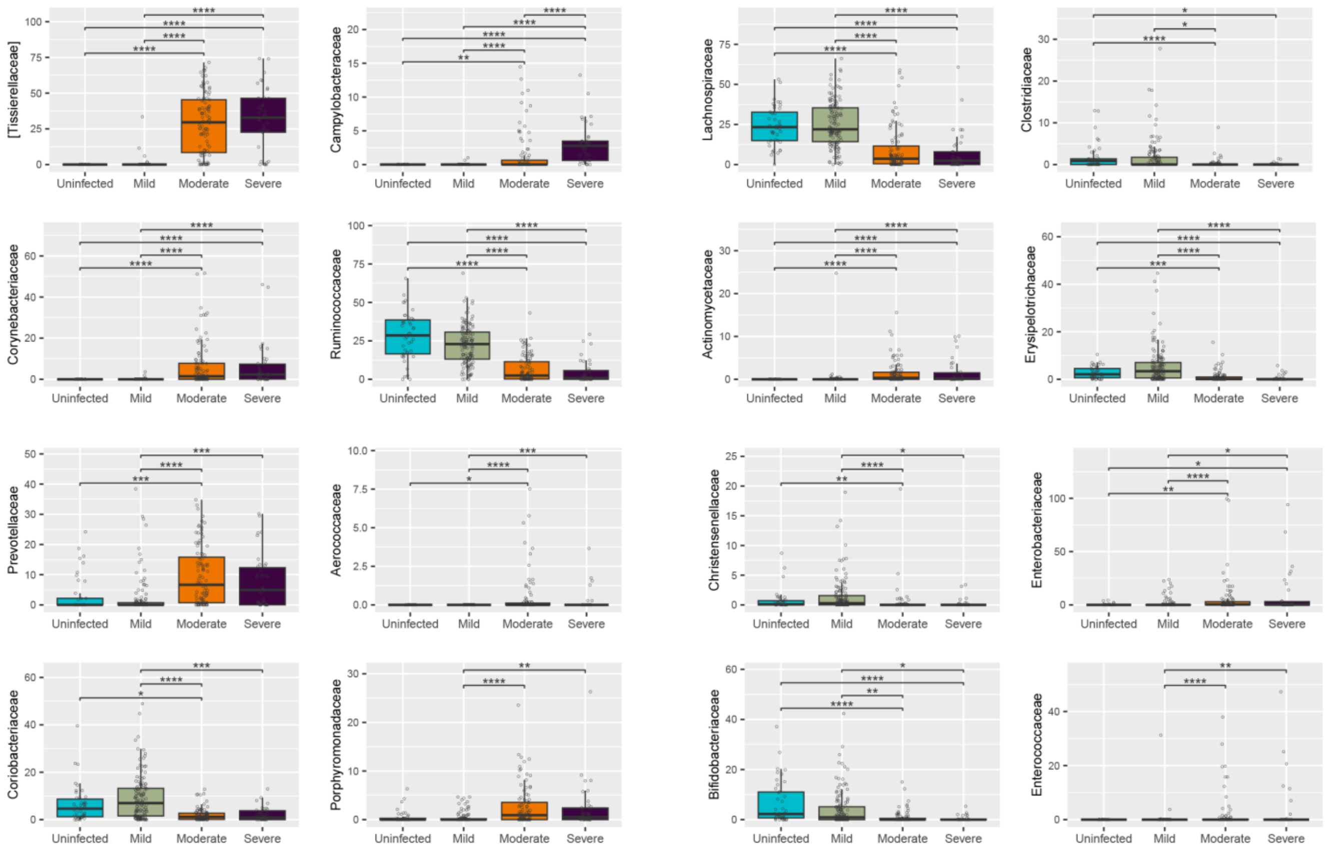

Supplementary Figure 2. Family-level differences in gut microbiota related to COVID-19 severity.

Boxplots showing the relative abundance distribution of families differentially represented between COVID-19 severity classes (uninfected, mild, moderate, severe). Significance bars indicate *p* values of Bonferroni-corrected pairwise Wilcoxon tests: *, $p < 0.05$; **, $p < 0.01$; ***, $p < 0.001$; ****, $p < 0.0001$.

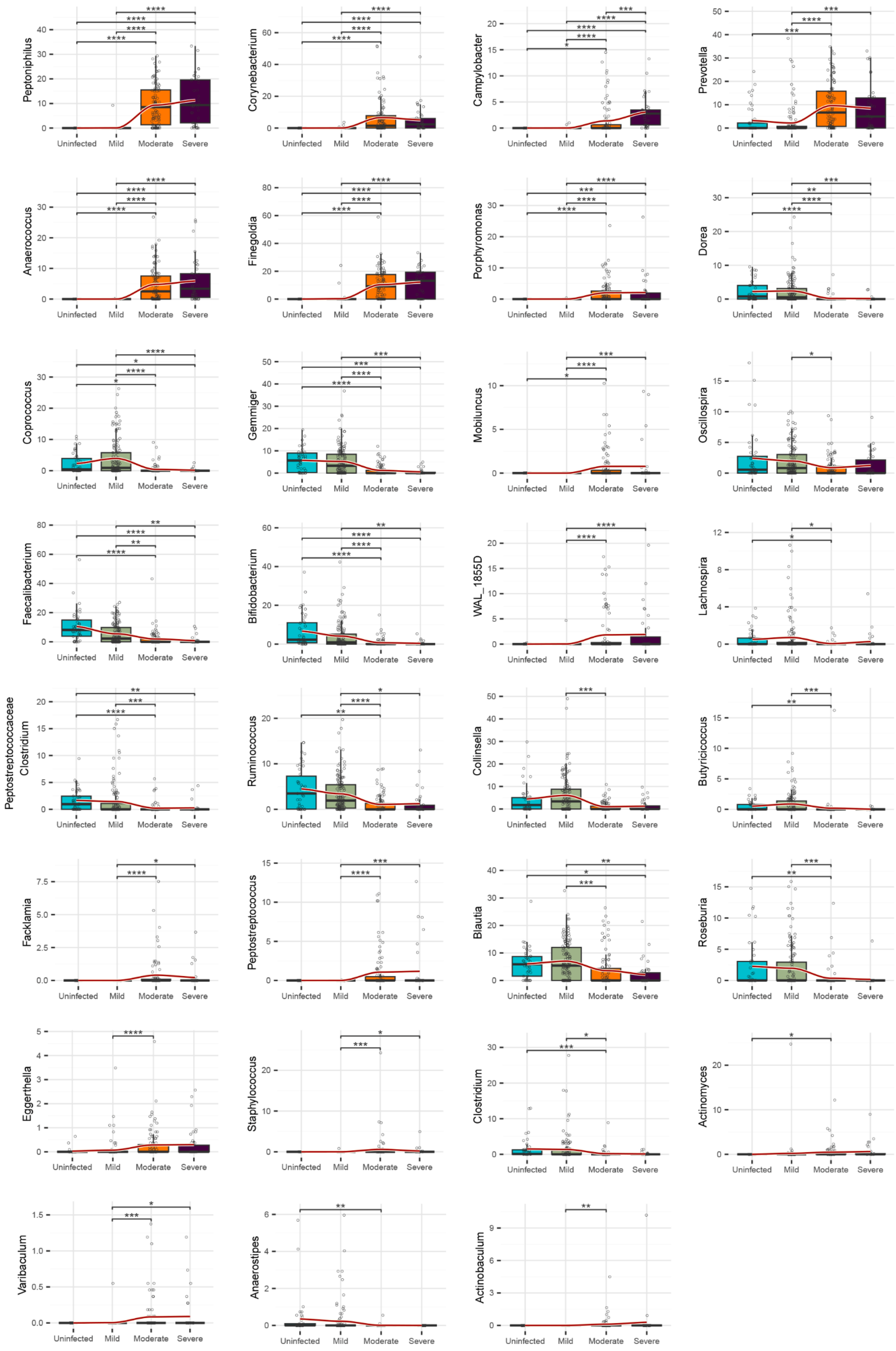

Supplementary Figure 3. Genus-level differences in gut microbiota related to COVID-19 severity.

Boxplots showing the relative abundance distribution of genera differentially represented between COVID-19 severity classes (uninfected, mild, moderate, severe). Smooth curve relationship estimates are also shown. Significance bars indicate *p* values of Bonferroni-corrected pairwise Wilcoxon tests: *, $p < 0.05$; **, $p < 0.01$; ***, $p < 0.001$; ****, $p < 0.0001$.

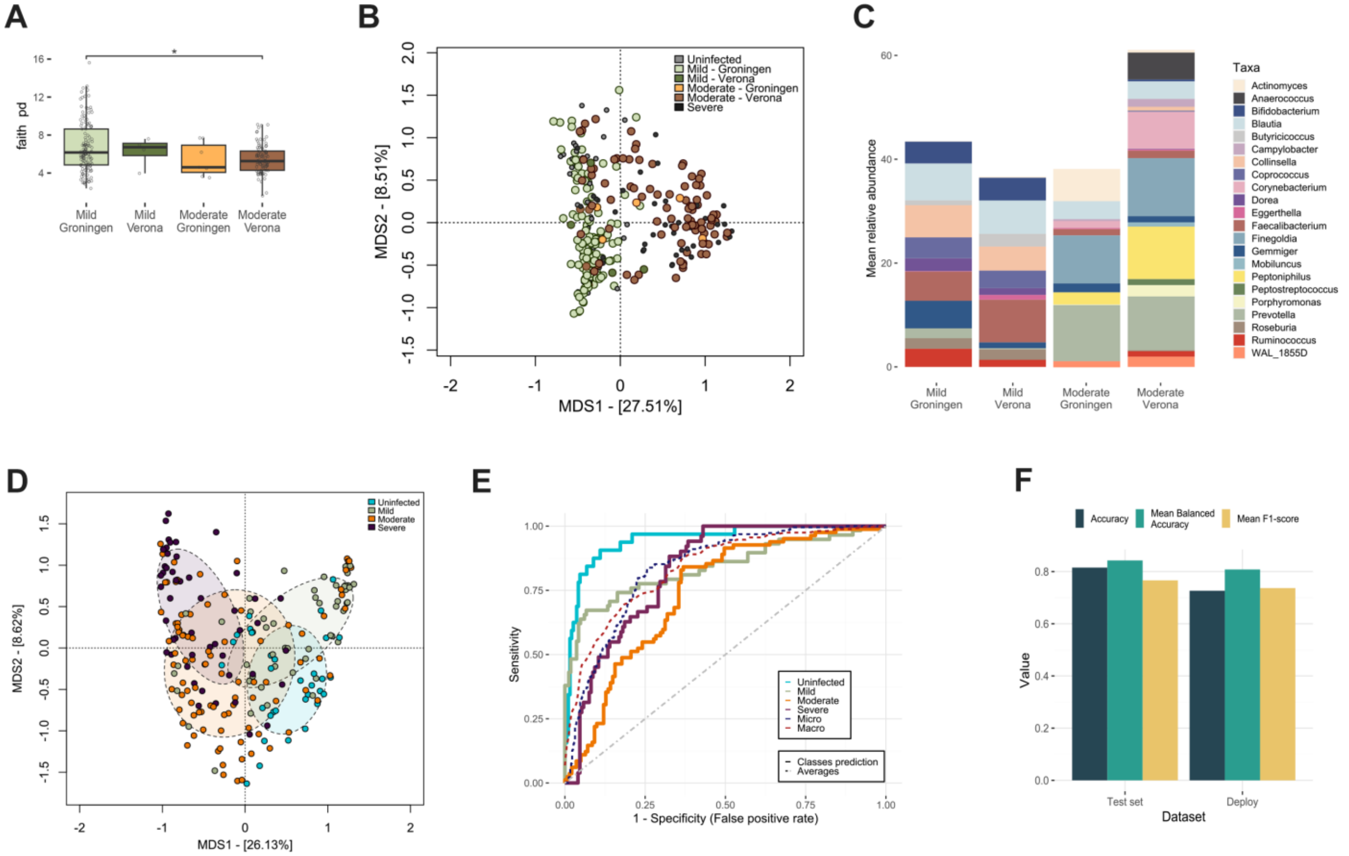

Supplementary Figure 4. Cohort biases evaluation and model deploy on external data.

(**A**) Boxplots display Faith's phylogenetic alpha diversity values, comparing Mild and Moderate severity classes in the Groningen and Verona cohorts. No significant cohort-related differences were detected using pairwise Wilcoxon tests. The only significant difference appears between the two severity classes as reported in **Figure 2A**. (**B**) Principal Coordinates Analysis (PCoA) of beta diversity is presented, computed with Unweighted UniFrac distances. Emphasis is given to the intra-Mild and intra-Moderate distribution of samples across both cohorts. (**C**) Barplots illustrate the average relative abundance values of genera highlighted via feature selection (as shown in Figure 2D). The representation is based on severity class and cohort. (**D**) PCoA of the external data samples according to the severity class. PERMANOVA differences are significant between groups similarly to what detected in our study. Ellipses represent the 95% confidence area based on the standard error of the weighted average of sample coordinates. (**E**) Receiver Operating Characteristic (ROC) curve depicts the prediction of severity classes on external datasets (model deployment). The dotted line represents micro and macro averages. (**F**) Model performance is compared between the test set (cohorts presented in this work) and the deploy dataset (external data). Metrics include accuracy, mean balanced accuracy, and mean F1-score.
